# Mitochondria Endoplasmic Reticulum Contact Sites (MERCs): Proximity Ligation Assay as a Tool to Study Organelle Interaction

**DOI:** 10.3389/fcell.2021.789959

**Published:** 2021-12-03

**Authors:** Sara Benhammouda, Anjali Vishwakarma, Priya Gatti, Marc Germain

**Affiliations:** ^1^ Groupe de Recherche en Signalisation Cellulaire and Département de Biologie Médicale, Université Du Québec à Trois-Rivières, Trois-Rivières, QC, Canada; ^2^ Centre D'Excellence en Recherche sur les Maladies Orphelines - Fondation Courtois, Université du Québec à Montréal, Montréal, QC, Canada

**Keywords:** mitochondia, endoplasmic reticulum, organelle contact sites, organelle, contact sites methodologies

## Abstract

Organelles cooperate with each other to regulate vital cellular homoeostatic functions. This occurs through the formation of close connections through membrane contact sites. Mitochondria-Endoplasmic-Reticulum (ER) contact sites (MERCS) are one of such contact sites that regulate numerous biological processes by controlling calcium and metabolic homeostasis. However, the extent to which contact sites shape cellular biology and the underlying mechanisms remain to be fully elucidated. A number of biochemical and imaging approaches have been established to address these questions, resulting in the identification of a number of molecular tethers between mitochondria and the ER. Among these techniques, fluorescence-based imaging is widely used, including analysing signal overlap between two organelles and more selective techniques such as *in-situ* proximity ligation assay (PLA). While these two techniques allow the detection of endogenous proteins, preventing some problems associated with techniques relying on overexpression (FRET, split fluorescence probes), they come with their own issues. In addition, proper image analysis is required to minimise potential artefacts associated with these methods. In this review, we discuss the protocols and outline the limitations of fluorescence-based approaches used to assess MERCs using endogenous proteins.

## Introduction

Organelles are responsible for many of the anabolic and catabolic processes required for the proper functioning of eukaryotic cells. For decades, organelle research has centred on identifying each compartment and their distinct properties with the thought that transfer of material between organelles occurred through diffusion of soluble metabolites or vesicular trafficking (Dennis and Kennedy, 1972). However, in recent years, the subject has undergone a revolution as we realised that cells use a network of contact sites between membranes of different organelles, termed membrane contact sites (MCS), to communicate and transfer metabolites (Schrader et al., 2015; Cohen et al., 2018; Scorrano et al., 2019). MCS are defined as areas of close apposition (typically less than 30 nm) between two organelles in the absence of membrane fusion. It is becoming clear that most, if not all, organelles interact via MCS. In addition, a growing number of proteins have been shown to be required for these MCS (Eisenberg-Bord et al., 2016; Simmen and Tagaya, 2017; Cohen et al., 2018; Scorrano et al., 2019).

Mitochondria-Endoplasmic-Reticulum (ER) contact sites (MERCS) play a central role in calcium signalling (Ca^2+^), phospholipid synthesis and transfer, regulation of oxidative stress and inflammatory responses, mitochondrial dynamics, bioenergetic and cell survival (Tubbs and Rieusset, 2017) (Patergnani et al., 2011; Krols et al., 2016; Martinvalet, 2018; Missiroli et al., 2018; Perrone et al., 2020; Xu et al., 2020). MERCS require proteins on both the ER and mitochondria to bridge the two organelles. While the nature of these protein tethers has not been fully characterized and likely varies depending on cellular conditions and cell types, several MERCS tethering complexes have been identified. This includes the interaction between ER-resident Inositol 1,4,5-triphosphate receptor (IP3R) and mitochondrial voltage-gated anion channel (VDAC) that is bridged by glucose-regulated protein 75 (GRP75), and the mitochondrial fusion protein Mitofusin-2 (MFN2) which localizes to both the ER and mitochondria (De Brito and Scorrano, 2008; Basso et al., 2018). Vesicle-associated membrane protein B (VAPB) and protein tyrosine phosphatase interacting protein 51 (PTPIP51) are other reported ER-mitochondria tethering proteins (Gomez-Suaga et al., 2017).

The size of a MERCS area and the width of the gap between the two organelles are critical structural characteristics of MERCS that are tightly regulated. While the underlying mechanisms are still being elucidated, it is clear that disruption of MERCS structure and/or activity is a crucial factor that promotes or contributes to oncogenesis, neurodegeneration, and a variety of other diseases (Kisby et al., 2011; López-Crisosto et al., 2015; Rodríguez-Arribas et al., 2017; Annunziata et al., 2018; Pinton, 2018; Eysert et al., 2020; Gao et al., 2020). Several imaging techniques have been used to study MERCS and, in general, MCS (recently reviewed by (Giamogante et al., 2020). These techniques include electron microscopy (EM), the gold standard for MCS identification, and fluorescence-based techniques that, while having a lower resolution, allow a more dynamic assessment of MCS and MERCS. Here, we will briefly review these techniques, focussing on proximity ligation assay (PLA), one of the most recent techniques used to study MERCS.

## Techniques for ER-Mitochondria Interaction

### Electron Microscopy

EM is the oldest tool for morphological examination of intracellular structures and provided the first evidence that ER and mitochondria interact (Copeland and Dalton, 1959). It allows the measure of both the distance between the organelles and the length of the extension. Also, EM provides strong membrane contrast and nanometer-scale resolution for observing cellular organelles. EM can also be combined with light microscopy using correlative light-electron microscopy (CLEM). Combining CLEM and 3D electron microscopy can also enhance the detailed structural studies of mitochondria and ER (Jung and Mun, 2019). Yet, EM analysis is extremely time-consuming. In addition, the fundamental restriction of EM is tissue fixation, which prohibits live cell imaging and monitoring MERCS dynamics. Another serious challenge with EM is quantifying apparent changes in organelle morphology. Point counting can be used to analyse mitochondria or ER morphology (Howard, 2004) but it has limited ability to quantify finer structural elements such as mito-ER interactions and cristae measurements. A simple method has recently been described using open source software ImageJ (Lam et al., 2021), but EM analysis remains time-consuming. Overall, while EM remains the gold standard for quantifying ER-mitochondria interaction, several techniques based on fluorescence have been developed to circumvent the limitation of EM.

### Fluorescent Microscopy

Fluorescence-based techniques to study MERCS are based on the use of antibodies that recognize the proteins of interest or the expression of proteins genetically tagged with Green Fluorescent Protein (GFP) or other fluorescent proteins and are easily available. The use of a combination of fluorescent-tagged proteins with distinct emission spectrums also allows dynamic interactions to be resolved in real time (Huang et al., 2020).

The simplest approach with fluorescence-based microscopy is to measure the co-localisation/overlap of fluorescent signals from cells co-transfected with fluorescence proteins targeted to ER and mitochondria. This has been used to demonstrate that ER-mitochondria interactions regulate calcium signaling (Rizzuto et al., 1998) and that mitochondrial fission occurs at MERCS (Friedman et al., 2011). The key advantages of this technology are its simplicity, fast processing, and compatibility with live imaging. The primary constraint of this technique is the resolution, which significantly limits our ability to distinguish organelles that are close to each other from those that actually interact. The use of live cell imaging can partially alleviate this by measuring the coordinated movement of the organelles on both sides of the MCS (Yang et al., 2018) or a functional consequence of this interaction (for example mitochondrial fission in the case of MERCS (Friedman et al., 2011)). Alternatively, it is possible to use a super-resolution fluorescence microscope that transcends traditional epi-fluorescence and confocal microscopy diffraction limits (Giamogante et al., 2020). Super-resolution microscopy can also be amenable to live cell imaging but requires a highly specialized microscope.

Generally, fluorescent images are processed to conduct a colocalization analysis that measures the amount of signal overlap between the two tagged organelles. While this colocalization does not necessarily represent the actual colocalization of two probes due to resolution limits, it can provide a useful estimate of changes occurring under the experimental conditions tested. To address this, most image processing software includes specific correlation measures: Pearson correlation coefficient (PCC) and Manders’ coefficients (Adler and Parmryd, 2010).

PCC measures how well the variation in pixel intensities between two signals can be explained by a simple, linear correlation between the two. As such, PCC is appropriate to measure the colocalization of two probes localized to the same cellular structure. However, the situation is more complex when measuring MCS because only a fraction of the signal is correlated (the MCS). In such situations, PCC measurements are ambiguous, if not misleading (Dunn et al., 2011). Manders’ coefficients provide an alternative to PCC by considering the co-occurrence of the signals (the fraction of pixels with positive values for both channels) independently of relative pixel intensity. There are two types of Manders’ coefficient: Manders’ overlap coefficient, which provides a global estimate of the overlap between the two signals akin to the PCC, and Manders’ colocalization coefficients that specifically assess the fraction of signal 1 overlapping with signal 2 and the fraction of signal 2 overlapping with signal 1. These coefficients can provide a useful measure of colocalization but require the proper determination of what constitutes the signal to measure, which is not trivial. Different strategies can be used to eliminate background signal, but these usually involve applying a global threshold to the image. Importantly, none of these methods (including the Costes automated method that is usually used when calculating Manders’ coeffficients) are effective in all situations. The selected strategy (global thresholding, local thresholding, use of preprocessing tools such as ImageJ’s Tubeness (Almutawa et al., 2019), etc.) needs to be carefully selected and validated so that the pixels that are selected as signal correspond to the actual signal. Changes in organelle structure can also affect Manders’ coefficients, as exemplified by the conflicting role of MFN2 in MERCS regulation as determined by fluorescence microscopy and EM (De Brito and Scorrano, 2008; Cosson et al., 2012; Filadi et al., 2015). A thorough discussion of the limitations of PCC and Manders’ coefficients can be found in (Dunn et al., 2011).

### Other Fluorescent Techniques

While the analysis of tagged organelles described above provides a rapid assessment of organelle colocalization that can be used to measure the dynamic interaction between organelles, its limitations have led to the development of more specific methods to probe MCS. These involve the transfection of fluorescent probes that can be detected only when two organelles are in close proximity or the use of antibodies for two distinct proteins that will be detected only when in close proximity.

Fluorescence-based techniques to selectively detect MCS are based on energy transfer (Förster energy transfer (FRET), bioluminescence resonance emission transfer (BRET)) or biomolecular fluorescence complementation (BiFC). FRET consists of energy transfer from an excitable fluorophore donor to a suited fluorophore acceptor (Shrestha et al., 2015). BRET provides an alternative to FRET where the donor fluorophore is replaced by luciferase which serves as the source of light for energy transfer to the acceptor (Pfleger and Eidne, 2006). In either case, donor/acceptor pairs are fused to resident ER and mitochondrial proteins and used to detect MERCS in an interaction-free approach (Naon et al., 2016). While FRET and BRET measure the energy transfer between to probes in close proximity, BiFC is based on the reconstitution of two fragments of a fluorescent protein (or luciferase) into a functional fluorophore. This technique requires the targeting of each fragment of the protein to a distinct organelle. Fluorescence is then observed at sites where the two organelles are in close proximity (MCS). In addition, because BiFC components have to assemble across membranes to be active, they can possibly promote MCS assembly.

### *In situ* Proximity Ligation Assay

Most of the approaches cited above require the overexpression of tagged proteins which is not amenable to all experimental systems. In addition, the overexpressed proteins can potentially affect the behaviour of target organelles. A highly sensitive approach recently being used to investigate endogenous protein interactions is *in situ* Proximity Ligation Assay (PLA) (Söderberg et al., 2006). This is a probe-based method in which endogenous proteins of interest are targeted by primary antibodies, followed by secondary antibodies fused to oligonucleotides. When the proteins are in close proximity, there is complementary base paring and the creation of circular DNA when a third oligonucleotide is added. The circular DNA can then be amplified and visualised using complementary fluorophore-labeled probes. PLA has a reported detection range of 40–60 nm, which is larger than the size of most MERCS. Nonetheless, this is greater than the resolution obtained by a typical fluorescence microscope and simple co-localisation analysis. The method’s advantage are its robustness and relative simplicity, as commercial kits are accessible (Söderberg et al., 2006; Hegazy et al., 2020). PLA also has the advantage of not requiring the expression of tagged proteins. However, it cannot be used in live cells and is limited by the availability of good antibodies for the proteins of interest.

### ER-Mitochondria Contact Assessment Using PLA

Though the PLA technique was established in 2006, its application in the investigation of MERCS emerged extensively in the last decade. ER-mitochondrial coupling was shown by PLA using several pairs of proteins (VDAC/IP3R, Grp75/IP3R, and CypD/IP3R) (Tubbs et al., 2014), while overexpression of VAPB (ER) and PTPIP51 (mitochondria) altered MERCS assembly, facilitating Ca^2+^ exchange and autophagy. (Gomez-Suaga et al., 2017). Furthermore, PLA has been widely utilized to study ER-mitochondria interactions in disease. Decreased MERCS were observed in cardiomyopathy caused by phopsholamban p. Arg14del mutation (Cuello et al., 2021), in Charcot-Marie-Tooth type 2A (CMT2A, a dominant axonal form of peripheral neuropathy due to mutation in MFN2 (Bernard-Marissal et al., 2019)) and also in conditions such as amyotrophic lateral sclerosis and frontal dementia (ALS/FTD) due to defects in fused in sarcoma (FUS) (Stoica et al., 2016). In addition, overexpression of α-Synuclein, a protein that accumulates in patients with Parkinson’s disease, disrupts binding between tethering proteins VAPB and PTPIP5 at MERCS (Paillusson et al., 2017). Overall, while supporting an important role for MERCS in cell physiology, the studies highlighted the usefulness of PLA in the study of MERCS.

### Considerations for the Analysis of PLA Foci

PLA has several advantages: 1) It allows the measurement of endogenous proteins at MERCs. 2) It provides dual-binder specificity for detecting organelle contacts *in situ* and exposes protein proximity in normal cells without being influenced by overexpression artefacts. (Söderberg et al., 2006). 3) Because the *in-situ* PLA signal can be amplified, the approach is extremely sensitive, allowing transient and weak interactions to be viewed and quantified as a single spot; and 4) The tool itself and its analysis (by examining the number of interacting spots) is very simple to perform and may be used to test multiple conditions.

**FIGURE 1:**
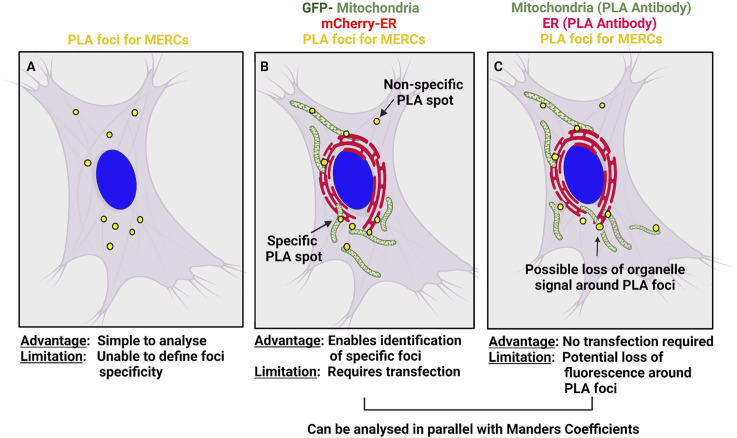
Analysis of PLA foci along with organelle markers. **(A)** Classical analysis of PLA foci where colocalization with organelle markers is not taken into consideration. **(B–C)** Alternatively, ER and mitochondria can be co-stained with PLA foci to further validate that PLA foci are localized at MERCS. This can be achieved using fluorescent-tagged proteins or stains **(B)** or fluorescent secondary antibodies recognizing the primary PLA antibodies.

Nevertheless, PLA has several shortcomings in addition to requiring fixed cells. The common procedure for studying the interaction of organelles is to count the number of PLA spots (Figure 1A). However, while ER or mitochondrial structures are easy to identify by immunofluorescence, the foci produced by the PLA signal can potentially be difficult to separate from non-specific signal. Non-specific PLA signal can come from two sources, the PLA procedure itself and the primary antibodies used. Isotype antibody controls are usually used to detect background fluorescence related to the PLA procedure. Further, to avoid background signals created by non-bound probes in close vicinity, the concentration of proximity probes must be kept low (Weibrecht et al., 2010; Jalili et al., 2018).

A second approach to validate PLA spots is to stain the organelles of interest along with PLA foci (Figures 1B,C). This allows to colocalize PLA spots with sites of overlap between the two organelles (Alpy et al., 2013; Gomez-Suaga et al., 2017; Sharma et al., 2021), thus identifying real PLA foci and allowing further assessment of organelle colocalization using Mander’s coefficients. Co-staining of PLA and organelles can be achieved by expressing fluorescently tagged organelles markers, using fixable stains such as mitotracker or labelling the primary antibodies used for the PLA. A large array of ER- or mitochondria-targeted fluorescent proteins (GFP, mCherry, etc.) as well as some fixable mitochondria stains (mitotracker) are readily available and can be used to label organelles prior fixation and PLA (Figure 1B). However, it does require cells to be transfected, which is not always possible.

Alternatively, the primary antibodies chosen to produce PLA spots can simply be labeled with fluorescent secondary antibodies, which will allow visualization of the organelles in addition to PLA foci and thus facilitate colocalization studies (Figure 1C). However, the presence of the amplified circular DNA could locally prevent the recognition of the primary antibodies by the fluorescent secondary antibodies. Nonetheless, examining for the presence and absence of organelles in the vicinity of the signal location will ensure that the PLA spots correspond to true contact sites.

Obtaining a clear, distinct, and quantifiable PLA signal also requires using quality antibodies that bind only the organelles of interest without background staining. It is thus critical to validate the antibodies used for PLA by first co-marking them with proper mitochondrial or ER markers (antibodies of fluorescent proteins) and only use antibodies that show highly specific staining of the organelle of interest. The choice of good PLA antibodies for the detection of MERCS can also be extended to proteins present within the contact site but that do not necessarily form a tethering pair. This is because PLA defines the proximity of the proteins, not necessarily their physical interaction. This means that while most PLA studies use protein pairs that have been shown to physically interact (for example VDAC1-IP3R), other pairs including membrane proteins could also be selected based on different criteria (i.e. availability of good antibodies).

## Conclusion

MERCs are signalling hubs consisting of structural components that play a critical role in a variety of pathways, ranging from the regulation of organelle homeostasis to a variety of cellular activities or signalling pathways, all of which ultimately affect cellular metabolism. In the last decade, the recognition of MERCS and more generally MCS as crucial controllers of cellular functions has led to the application of novel tools to study organelle interaction. These methods have significantly expedited recent developments in the field but come with their drawbacks. PLA in particular has the potential to simplify the quantification of MCS but requires careful validation of the antibodies used and the result. Ultimately, it is important to validate results using different approaches that investigate MERCS functions (like calcium transport) or performing complementary biochemical, fluorescent, and EM approaches.

## References

[B1] Adler J. Parmryd I. (2010). Quantifying Colocalization by Correlation: the Pearson Correlation Coefficient Is superior to the Mander′s Overlap Coefficient. Cytometry 77A, 733–742. 10.1002/cyto.a.20896 20653013

[B2] Almutawa W. Smith C. Sabouny R. Smit R. B. Zhao T. Wong R. (2019). The R941L Mutation in MYH14 Disrupts Mitochondrial Fission and Associates with Peripheral Neuropathy. EBioMedicine 45, 379–392. 10.1016/j.ebiom.2019.06.018 31231018 PMC6642256

[B3] Alpy F. Rousseau A. Schwab Y. Legueux F. Stoll I. Wendling C. (2013). STARD3 or STARD3NL and VAP Form a Novel Molecular Tether between Late Endosomes and the ER. J. Cel Sci 126, 5500–5512. 10.1242/jcs.139295 24105263

[B4] Annunziata I. Sano R. d’Azzo A. (2018). Mitochondria-associated ER Membranes (MAMs) and Lysosomal Storage Diseases. Cell Death Dis 9, 328. 10.1038/s41419-017-0025-4 29491402 PMC5832421

[B5] Basso V. Marchesan E. Peggion C. Chakraborty J. Von Stockum S. Giacomello M. (2018). Regulation of ER-Mitochondria Contacts by Parkin via Mfn2. Pharmacol. Res. 138, 43–56. 10.1016/j.phrs.2018.09.006 30219582

[B6] Bernard-Marissal N. Van Hameren G. Juneja M. Pellegrino C. Louhivuori L. Bartesaghi L. (2019). Altered Interplay between Endoplasmic Reticulum and Mitochondria in Charcot-Marie-Tooth Type 2A Neuropathy. Proc. Natl. Acad. Sci. USA 116, 2328–2337. 10.1073/pnas.1810932116 30659145 PMC6369737

[B7] Cohen S. Valm A. M. Lippincott-Schwartz J. (2018). Interacting Organelles. Curr. Opin. Cel Biol. 53, 84–91. 10.1016/j.ceb.2018.06.003 PMC6241252 30006038

[B8] Copeland D. E. Dalton A. J. (1959). An Association between Mitochondria and the Endoplasmic Reticulum in Cells of the Pseudobranch Gland of a Teleost. J. Biophys. Biochem. Cytol. 5, 393–396. 10.1083/jcb.5.3.393 13664679 PMC2224680

[B9] Cosson P. Marchetti A. Ravazzola M. Orci L. (2012). Mitofusin-2 Independent Juxtaposition of Endoplasmic Reticulum and Mitochondria: an Ultrastructural Study. PLoS One 7, e46293. 10.1371/journal.pone.0046293 23029466 PMC3460865

[B10] Cuello F. Knaust Ae. Saleem U. Loos M. Raabe J. Mosqueira D. (2021). Impairment of the ER/mitochondria compartment in human cardiomyocytes with PLN p.Arg14del mutation. EMBO Mol. Med. 13, e13074. 10.15252/emmm.202013074 33998164 PMC8185541

[B11] De Brito O. M. Scorrano L. (2008). Mitofusin 2 Tethers Endoplasmic Reticulum to Mitochondria. Nature 456, 605–610. 10.1038/nature07534 19052620

[B12] Dennis E. A. Kennedy E. P. (1972). Intracellular Sites of Lipid Synthesis and the Biogenesis of Mitochondria. J. Lipid Res. 13, 263–267. 10.1016/s0022-2275(20)39421-9 5016308

[B13] Dunn K. W. Kamocka M. M. Mcdonald J. H. (2011). A Practical Guide to Evaluating Colocalization in Biological Microscopy. Am. J. Physiology-Cell Physiol. 300, C723–C742. 10.1152/ajpcell.00462.2010 PMC3074624 21209361

[B14] Eisenberg-Bord M. Shai N. Schuldiner M. Bohnert M. (2016). A Tether Is a Tether Is a Tether: Tethering at Membrane Contact Sites. Dev. Cel 39, 395–409. 10.1016/j.devcel.2016.10.022 27875684

[B15] Eysert F. Kinoshita Pf. Mary A. Vaillant-Beuchot L. Checler F. Chami M. (2020). Molecular Dysfunctions of Mitochondria-Associated Membranes (MAMs) in Alzheimer's Disease. Int. J. Mol. Sci. 21, 9521. 10.3390/ijms21249521 PMC7765134 33327665

[B16] Filadi R. Greotti E. Turacchio G. Luini A. Pozzan T. Pizzo P. (2015). Mitofusin 2 Ablation Increases Endoplasmic Reticulum-Mitochondria Coupling. Proc. Natl. Acad. Sci. USA 112, E2174–E2181. 10.1073/pnas.1504880112 25870285 PMC4418914

[B17] Friedman J. R. Lackner L. L. West M. Dibenedetto J. R. Nunnari J. Voeltz G. K. (2011). ER Tubules Mark Sites of Mitochondrial Division. Science 334, 358–362. 10.1126/science.1207385 21885730 PMC3366560

[B18] Gao P. Yang W. Sun L. (2020). Mitochondria-Associated Endoplasmic Reticulum Membranes (MAMs) and Their Prospective Roles in Kidney Disease. Oxid Med. Cel Longev 2020, 3120539. 10.1155/2020/3120539 PMC7487091 32952849

[B19] Giamogante F. Barazzuol L. Brini M. Calì T. (2020). ER-mitochondria Contact Sites Reporters: Strengths and Weaknesses of the Available Approaches. Int. J. Mol. Sci. 21, 8157. 10.3390/ijms21218157 PMC7663704 33142798

[B20] Gomez-Suaga P. Paillusson S. Stoica R. Noble W. Hanger D. P. Miller C. C. J. (2017). The ER-Mitochondria Tethering Complex VAPB-PTPIP51 Regulates Autophagy. Curr. Biol. 27, 371–385. 10.1016/j.cub.2016.12.038 28132811 PMC5300905

[B21] Hegazy M. Cohen-Barak E. Koetsier Jl. Najor Na. Arvanitis C. Sprecher E. (2020). Proximity Ligation Assay for Detecting Protein-Protein Interactions and Protein Modifications in Cells and Tissues *In Situ* . Curr. Protoc. Cel Biol 89, e115. 10.1002/cpcb.115 PMC8041061 33044803

[B22] Howard V. R. M. (2004). Unbiased Stereology: Three-Dimensional Measurement in Microscopy. New York, NY, USA: Garland Science.

[B23] Huang X. Jiang C. Yu L. Yang A. (2020). Current and Emerging Approaches for Studying Inter-organelle Membrane Contact Sites. Front. Cel Dev. Biol. 8, 195. 10.3389/fcell.2020.00195 PMC7118198 32292782

[B24] Jalili R. Horecka J. Swartz J. R. Davis R. W. Persson H. H. J. (2018). Streamlined Circular Proximity Ligation Assay Provides High Stringency and Compatibility with Low-Affinity Antibodies. Proc. Natl. Acad. Sci. USA 115, E925–E933. 10.1073/pnas.1718283115 29339495 PMC5798375

[B25] Jung M. Mun J. Y. (2019). Mitochondria and Endoplasmic Reticulum Imaging by Correlative Light and Volume Electron Microscopy. J. Vis. Exp 149, e59750. 10.3791/59750 31380837

[B26] Kisby G. E. Fry R. C. Lasarev M. R. Bammler T. K. Beyer R. P. Churchwell M. (2011). The Cycad Genotoxin MAM Modulates Brain Cellular Pathways Involved in Neurodegenerative Disease and Cancer in a DNA Damage-Linked Manner. PLoS One 6, e20911. 10.1371/journal.pone.0020911 21731631 PMC3121718

[B27] Krols M. Bultynck G. Janssens S. (2016). ER-mitochondria Contact Sites: A New Regulator of Cellular Calcium Flux Comes into Play. J. Cel Biol 214, 367–370. 10.1083/jcb.201607124 PMC4987300 27528654

[B28] Lam J. Katti P. Biete M. Mungai M. Ashshareef S. Neikirk K. (2021). A Universal Approach to Analyzing Transmission Electron Microscopy with ImageJ. Cells 10, 2177. 10.3390/cells10092177 34571826 PMC8465115

[B29] López-Crisosto C. Bravo-Sagua R. Rodriguez-Peña M. Mera C. Castro P. F. Quest A. F. G. (2015). ER-to-mitochondria Miscommunication and Metabolic Diseases. Biochim. Biophys. Acta (Bba) - Mol. Basis Dis. 1852, 2096–2105. 10.1016/j.bbadis.2015.07.011 26171812

[B30] Martinvalet D. (2018). The Role of the Mitochondria and the Endoplasmic Reticulum Contact Sites in the Development of the Immune Responses. Cel Death Dis 9, 336. 10.1038/s41419-017-0237-7 PMC5832423 29491398

[B31] Missiroli S. Patergnani S. Caroccia N. Pedriali G. Perrone M. Previati M. (2018). Mitochondria-associated Membranes (MAMs) and Inflammation. Cel Death Dis 9, 329. 10.1038/s41419-017-0027-2 PMC5832426 29491386

[B32] Naon D. Zaninello M. Giacomello M. Varanita T. Grespi F. Lakshminaranayan S. (2016). Critical Reappraisal Confirms that Mitofusin 2 Is an Endoplasmic Reticulum-Mitochondria Tether. Proc. Natl. Acad. Sci. USA 113, 11249–11254. 10.1073/pnas.1606786113 27647893 PMC5056088

[B33] Paillusson S. Gomez-Suaga P. Stoica R. Little D. Gissen P. Devine M. J. (2017). α-Synuclein Binds to the ER-Mitochondria Tethering Protein VAPB to Disrupt Ca2+ Homeostasis and Mitochondrial ATP Production. Acta Neuropathol. 134, 129–149. 10.1007/s00401-017-1704-z 28337542 PMC5486644

[B34] Patergnani S. Suski J. M. Agnoletto C. Bononi A. Bonora M. De Marchi E. (2011). Calcium Signaling Around Mitochondria Associated Membranes (MAMs). Cell Commun Signal 9, 19. 10.1186/1478-811x-9-19 21939514 PMC3198985

[B35] Perrone M. Caroccia N. Genovese I. Missiroli S. Modesti L. Pedriali G. (2020). The Role of Mitochondria-Associated Membranes in Cellular Homeostasis and Diseases. Int. Rev. Cel Mol Biol 350, 119–196. 10.1016/bs.ircmb.2019.11.002 32138899

[B36] Pfleger K. D. G. Eidne K. A. (2006). Illuminating Insights into Protein-Protein Interactions Using Bioluminescence Resonance Energy Transfer (BRET). Nat. Methods 3, 165–174. 10.1038/nmeth841 16489332

[B37] Pinton P. (2018). Mitochondria-associated Membranes (MAMs) and Pathologies. Cel Death Dis 9, 413. 10.1038/s41419-018-0424-1 PMC5856760 29549303

[B38] Rizzuto R. Pinton P. Carrington W. Fay F. S. Fogarty K. E. Lifshitz L. M. (1998). Close Contacts with the Endoplasmic Reticulum as Determinants of Mitochondrial Ca 2+ Responses. Science 280, 1763–1766. 10.1126/science.280.5370.1763 9624056

[B39] Rodríguez-Arribas M. Yakhine-Diop S. M. S. Pedro J. M. B.-S. Gómez-Suaga P. Gómez-Sánchez R. Martínez-Chacón G. (2017). Mitochondria-Associated Membranes (MAMs): Overview and its Role in Parkinson′s Disease. Mol. Neurobiol. 54, 6287–6303. 10.1007/s12035-016-0140-8 27714635

[B40] Schrader M. Godinho L. F. Costello J. L. Islinger M. (2015). The Different Facets of Organelle Interplay-An Overview of Organelle Interactions. Front. Cel Dev. Biol. 3, 56. 10.3389/fcell.2015.00056 PMC4585249 26442263

[B41] Scorrano L. De Matteis M. A. Emr S. Giordano F. Hajnóczky G. Kornmann B. (2019). Coming Together to Define Membrane Contact Sites. Nat. Commun. 10, 1287. 10.1038/s41467-019-09253-3 30894536 PMC6427007

[B42] Sharma G. Saubouny R. Joel M. M. Martens K. Martino D. De Koning A. P. J. (2021). Characterization of a Novel Variant in the HR1 Domain of MFN2 in a Patient with Ataxia, Optic Atrophy and Sensorineural Hearing Loss [version 1; peer review: 2 approved with reservations]. F1000Research 10 606 10.12688/f1000research.53230.1 PMC10808857 38274408

[B43] Shrestha D. Jenei A. Nagy P. Vereb G. Szöllősi J. (2015). Understanding FRET as a Research Tool for Cellular Studies. Ijms 16, 6718–6756. 10.3390/ijms16046718 25815593 PMC4424985

[B44] Simmen T. Tagaya M. (2017). Organelle Communication at Membrane Contact Sites (MCS): From Curiosity to Center Stage in Cell Biology and Biomedical Research. Adv. Exp. Med. Biol. 997, 1–12. 10.1007/978-981-10-4567-7_1 28815518

[B45] Söderberg O. Gullberg M. Jarvius M. Ridderstråle K. Leuchowius K.-J. Jarvius J. (2006). Direct Observation of Individual Endogenous Protein Complexes *In Situ* by Proximity Ligation. Nat. Methods 3, 995–1000. 10.1038/nmeth947 17072308

[B46] Stoica R. Paillusson S. Gomez‐Suaga P. Mitchell J. C. Lau D. H. Gray E. H. (2016). ALS/FTD ‐associated FUS Activates GSK ‐3β to Disrupt the VAPB - PTPIP 51 Interaction and ER -mitochondria Associations. EMBO Rep. 17, 1326–1342. 10.15252/embr.201541726 27418313 PMC5007559

[B47] Tubbs E. Rieusset J. (2017). Metabolic Signaling Functions of ER-Mitochondria Contact Sites: Role in Metabolic Diseases. J. Mol. Endocrinol. 58, R87–R106. 10.1530/jme-16-0189 27965371

[B48] Tubbs E. Theurey P. Vial G. Bendridi N. Bravard A. Chauvin M.-A. (2014). Mitochondria-associated Endoplasmic Reticulum Membrane (MAM) Integrity Is Required for Insulin Signaling and Is Implicated in Hepatic Insulin Resistance. Diabetes 63, 3279–3294. 10.2337/db13-1751 24947355

[B49] Weibrecht I. Leuchowius K.-J. Clausson C.-M. Conze T. Jarvius M. Howell W. M. (2010). Proximity Ligation Assays: a Recent Addition to the Proteomics Toolbox. Expert Rev. Proteomics 7, 401–409. 10.1586/epr.10.10 20536310

[B50] Xu L. Wang X. Tong C. (2020). Endoplasmic Reticulum-Mitochondria Contact Sites and Neurodegeneration. Front. Cel Dev. Biol. 8, 428. 10.3389/fcell.2020.00428 PMC7314981 32626703

[B51] Yang Z. Zhao X. Xu J. Shang W. Tong C. (2018). A Novel Fluorescent Reporter Detects Plastic Remodeling of Mitochondria-ER Contact Sites. J. Cel Sci 131, jcs208686. 10.1242/jcs.208686 29158224

